# Biological Properties of Essential Oils from *Thymus algeriensis* Boiss

**DOI:** 10.3390/plants10040786

**Published:** 2021-04-16

**Authors:** Hamza Ouakouak, Adel Benarfa, Mohammed Messaoudi, Samir Begaa, Barbara Sawicka, Naima Benchikha, Jesus Simal-Gandara

**Affiliations:** 1 Chemistry Department, University of Hamma Lakhdar, B.P.789, 39000 El-Oued, Algeria; ouakouak-hamza@univ-eloued.dz (H.O.); m.messaoudi@crnb.dz (M.M.); naima_chem@yahoo.fr (N.B.); 2 Technical Platform of Physico-Chemical Analysis (PTAPC-Laghouat-CRAPC), P.O. Box. 37G, Road of Ghardaïa, 03000 Laghouat, Algeria; adel.benarfa@gmail.com; 3 Nuclear Research Centre of Birine, P.O. Box 180, Ain Oussera, 17200 Djelfa, Algeria; 4 Department of Plant Production Technology and Commodities Science, University of Life Science in Lublin, Akademicka 15 str., 20-950 Lublin, Poland; barbara.sawicka@up.lublin.pl; 5 Nutrition and Bromatology Group, Department of Analytical and Food Chemistry, Faculty of Food Science and Technology, University of Vigo—Ourense Campus, E32004 Ourense, Spain

**Keywords:** *Thymus algeriensis* Boiss, essential oils, chemical composition, GC-MS, antitumor activity, antimicrobial activity, antioxidant activity

## Abstract

This study describes the chemical composition, antitumor, antioxidant, and antimicrobial activities of the plant *Thymus algeriensis* Boiss. Essential oils (EOs) were collected in different periods (before, during, and after flowering stage) from the El-Guetfa region, M’sila, Algeria. The EOs extraction was achieved using three distinguishing techniques: hydro (Clevenger trap), steam, and microwave distillations, targeting different aerial parts of the plant (stems, flowers, and leaves). The EOs chemical components were estimated using GC-FID and GC-MS apparatuses. The resulting yield of the extracted oil was moderate and ranged between 0.84 and 1.53% (*wt*/*vol*). In total, eighty-five components were identified, in which the oxygenated monoterpenes family formed the main portion, starting from 40.56 up to 70.66%. The obtained essential oil was dominated by five major components that varied from low to quite moderate percentages: camphor (17.45–32.56%), borneol (11.16–22.2%), camphene (7.53–12.86%), 1.8-cineole (5.16–11.21%), and bornyl acetate (3.86–7.92%). The biological results of this oil pointed out that the EOs extracted from the leaves part exposed a weak radical scavenging activity afterward using two well-known antioxidant assays DPPH (IC_50_ = 8.37 mg/mL) and ABTS (10.84 mg/mL). Meanwhile, this oil presented strong inhibition activity against colon cancer cell line HCT116 (LC50 = 39.8 µg/mL) and a moderate inhibitory against hepatocellular cancer cells HePG2 (LC50 > 100 µg/mL). In addition, this oil antimicrobial activity was quite important against *Micrococcus luteus (M. luteus*)*, Staphylococcus aureus* CIP 7625, *Escherichia coli* ATCC 10536, *Saccharomyces cerevisiae* ATCC *4226*, *Candida albicans* IPA200, *Candida tropicalis (Ct),* and *Candida glabrata (Cg)* after using Amoxicillin and Itraconazole as references.

## 1. Introduction

The importance of essential oils aromatherapy was known for thousands of years by ancient civilizations, and it is well known that ancient Chinese civilization was the founder and the first to use this kind of treatment [1]. By the turn of the 19th century, essential oils were widely used and subjected to scientific investigation, especially medicinal and cosmetic preparations. Nowadays, many researchers show that a lot of medicinal plant species’ essential oils possess numerous and different organic compounds such as terpenes, esters, alcohols, ketones, aldehydes, phenols, etc., which opens another window research regarding the manner of essential oil extraction and purifying the complexity mixtures of medicinal plant essential oils. In this context, numerous researchers have paid special consideration to natural antioxidant originating from plants, which possess a lot of bioactive elements present in slight minor amounts that act for instance as antivirals, bactericides, antioxidants, or more important antitumor compounds. 

Many studies have confirmed that EOs are a complex of volatile compounds with high biological activities [2,3,4]. EOs can be extracted from various parts of plants, such as roots, rhizome, tubers, leaves, stems, bark, buds, flowers, inflorescences, seeds, and fruits, depending on the species and variety [5,6]. Then, the recovered extracted EOs could be used in a wide variety of biological activities such as anti-cancer [7], antioxidant [8], anti-inflammatory [9] and antibacterial activities [6]. In addition, EOs improve the quality of the foods without residues in the product or the environment, so they are considered a safe alternative as food additives compared to synthetic agents [10].

Nowadays, several medicinal plants are consumed or used due to their medicinal properties as well as their nutritional value. Indeed, inhabitants of the world, including Algerians, believed that eating and consuming fresh medicinal plants may treat illnesses and ailments [11,12,13]; among those plants was *Thymus algeriensis* Boiss, which belongs to the *Lamiaceae* family [14]. In fact, this family is among the largest families of flowering plants with about 250 genera and over 7000 species distributed around the world. This species includes 11 botanical varieties (*Thymus algeriensis* var. *cinerascens* (Murb.) Maire; *Thymus algeriensis* var. *Maire masculensis*; *Thymus algeriensis* var. *pomelii* Maire; *Thymus algeriensis* var. *villicaulis* Maire; *Thymus ciliatus* subsp. *algeriensis* (Boiss. & amp; Reut.) Batt.; *Thymus ciliatus* var. *zattarellus* (Pomel) Batt.; *Thymus hirtus* subsp. *algeriensis* (Boiss. & amp; Reut.) Murb.; *Thymus hirtus* var. *battandieri* Ronniger & Sennen; *Thymus hirtus* var. *cinerascens* Murb.; *Thymus tunetanus* Pomel; *Thymus zattarellus* Pomel) [14]. These botanical varieties are considered as important sources of essential oils, which contain quite important chemical components: for example, menthol, geraniol, eucalyptol, camphor, and thymol [15].

In Algeria, the *Thymus algerian* Boiss. Reut. (Synonym Thymus hirtus Willd. Subsp. algeriensis Boiss. Et Reut.) plant, which known is as “Himria” in the Algerian vernacular language, is classified by the local inhabitants especially in rural communities as an important and indispensable element in healing some common age diseases such as stomach or gastrointestinal diseases and physiotherapy [16]. Moreover, this plant’s EOs are used to enhance the immune system and help fight colds, flu, infectious diseases, and chills [17]. In the present study, we tried to determine the chemical composition of *Thymus algeriensis* Boiss., essential oil, harvested in three different growing stages: before, during, and after the flowering plant period, targeting different parts from the plant (stems, flowers, and leaves), and using three distinguished extraction techniques, hydrodistillation (HD), steam distillation (SD), and microwave-assisted distillation (MAD) in order to maximize the extraction yield and see the differences in the resulted EOs chemical components. On the other half, some important *in vitro* and *in vivo* biological tests were performed on the recovered EOs, including the following:

− Antioxidant test using two well-known reagents or assays DPPH (2,2-diphenyl-1-picrylhydrazyl) and ABTS (2,2′-azino-bis (3-ethylbenzothiazoline-6-sulfonic acid);
− Antitumor test using two kinds of human cancer cells colon and hepatocellular carcinoma cells;
− A microbiol test using several strains (bacteria, yeast, and fungi).

Moreover, many cytotoxic activity tests of *Thymus algeriensis* EOs have been done on several tumor cells such as breast cancer MCF-7, small cell lung cancer NCI-H460, Colon cancer (HCT15), Cervical cancer (HeLa), Human Prostate Cancer (PC3), and Human leukemia (K562); those tests have given ineffective results compared to the reference compounds used (Doxorubicin and Elipticine) [17,18].

In parallel, our results against colon cancer cell line HCT116 showed a quite amazing results comparing with the same reference “Doxorubicin” used by the above-mentioned works. 

## 2. Results and Discussion 

### 2.1. Chemical Composition

**Figure 1:**
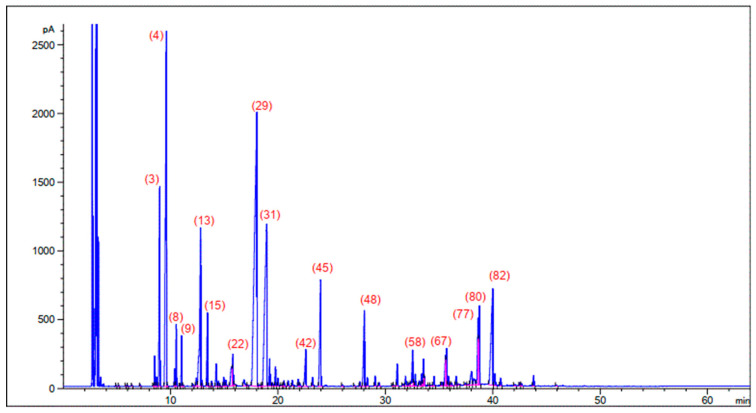
Chromatogram of GC-FID-H-aerial part-HD essential oils of *Thymus algeriensis* Boiss. The numbers in Figure 1 represent the numbering of the compounds shown in Table 1.

Through the integration between the two previous analyses (GC-FID and GC-MS techniques), the quantitative results were taken from the GC-FID technique analyses (see Figure 1), while the qualitative results were taken from the GC-MS technique. The results of extraction yield of the samples collected during the flowering plant stage, after using the hydrodistillation of both parts “all areal part” and “leaves”, were 1.30% and 1.53%. A total of eighty-five compounds were identified for both parts, which presented about 95.99 and 98.97% for areal parts and leaves samples, respectively.

It could be noticed from Table 1 that camphor, borneol, camphene, 1,8-cineol, and bornyl acetate were the major constituents, in addition to a much lower concentration of α pinene, limonene, cis-sabinene hydrate, terpinene-4-ol, isobornyl formate, β-bourbonene, alloaromadendrene, bicyclogermacrene, eudesmol, and cedranol. The oil from Thymus algeriensis has been proven to be rich in monoterpene hydrocarbons (≈14.32% to 26.90%), oxygenated monoterpenes (≈40.56% to 70.66%), sesquiterpene hydrocarbons (1.01% to 5.07%), and oxygenated sesquiterpene (2.84% to 17.04%).

The organic chemical of camphor has a strong odor; it also has antibacterial and antifungal properties that make it useful in healing infections. The lotions and creams containing camphor are famous for relieving skin irritation and itchiness and may help to improve the overall appearance of the skin. In addition, this chemical component is considered as an effective element to treat wounds and ultraviolet light-induced wrinkles, and it is used for anti-aging as well [19]. The concentration of camphor in the studied samples ranged from 17.45% to 34.31%, where a high presence (percentage) of this element was found in the aerial parts of the samples that were collected after the flowering stage of the plant.

The organic chemical borneol was used widely in Chinese traditional medicine as a drug, because it facilitates the transport of multiple drugs to specific sites [20]. It has been shown to be able to deliver drugs in the brain efficiently. Our results have shown that this compound can improve the efficiency of brain targeting of mixed nanoparticles, and it is also an anti-inflammatory [21]. The percentage of borneol obtained in the studied samples was from 11.16% to 17.13%, and it was noticed that this element percentage is less than that of camphor for the same targeted parts and period collection. 

Camphene is an organic chemical that is known to have a pungent smell; it works effectively in the treatment of fungal skin infections, dysentery, and foot and skin infections; it also treats bacterial, fungal, and viral infections that affect the respiratory system, it completely treats congestion, and it has also been used to treat acute respiratory disorders such as bronchitis [22]. It also works with vitamin C as an antioxidant and helps repair damage caused by stress [13]. These compounds work together and influence a calming effect on nerves, reducing blood pressure, inflammation, and stress [23,24]. In the studied sample, the content of this compound, was found to range between 12.78% and 14.88%, where its high percentage value was found in leaves during the flowering stage.

The measures of variability (dispersion) characterize the statistical community, considering the differences between the individual units that make it up. They characterize the degree of diversity of the community due to the distinguished variable features. The study uses both classic measures of variability, such as variance, standard deviation, and classical variation coefficient, as well as positional measures, such as range and skewness. The characteristics of the measures of the examined features are presented in Table 2. The feature that best describes the stability of the assessed quality features is the coefficient of variation, which is a relative measure of diversity, informing about the strength of diversity of the surveyed population in terms of the variable feature and enabling the evaluation of the arithmetic mean. The higher the coefficient value, the stronger the differentiation, and vice versa. The retention indexes turned out to be the most stable features, and the content of essential oils in the leaves marked during flowering was the most variable (Table 2).

Kurtosis is understood as the degree of clustering of individual units of the community due to the studied variable around the arithmetic mean of this variable, which means in this case the deformation of the distribution due to the flattening or slenderness of the abundance curve. The more slender the abundance curve, the stronger the concentration (degree of concentration), and the more flattened the abundance curve, the weaker the concentration (degree of concentration). In the case of the remediation index, the value of kurtosis was below 3.0, which means a flattened curve; in other cases, this value was >3, which means a slender curvature of the aggregation of these results. The measures of asymmetry (skewness) determined the direction of the distribution of the examined variable features in the community and the degree of deviation of the variable feature distribution from the symmetric distribution. The distribution of the examined features turned out to be asymmetric on the right side (positive asymmetry). Asymmetry means deformation of the distribution of the studied variables due to the extension of the abundance curve to the right in relation to the dominant. The bigger the asymmetry of the distribution, the smaller the cognitive value of the arithmetic mean and other classical measures (Table 2).

Retention indices did not depend on the methods and dates of obtaining the oils. The content of individual methods and dates of obtaining thyme oils turned out to be highly significantly related to each other (Table 3). This can be explained by the high internal correlation. The strength of the relationship between non-measurable variables can be better defined by the corrected contingency coefficient, the Chuprov coefficient, or the Cramer’s V-convergence coefficient, which are based on the chi-square test of independence [16]. These coefficients assume values in the range <0; 1>, where the value closer to 1 means that the dependence is stronger, while a value closer to 0 indicates a weaker dependence [25].

From this result, the best extraction method for oil production (leaves HD EOs) will be relied upon for use in the rest of the study with a comparison of the root scavenging of leaves and the aerial part EOs. The best way to obtain a good yield of oils in the case of obtaining this raw material from the aerial parts of thyme turned out to be extraction by hydrodistillation (HD) in the period after flowering of the plants; in the case of camphene harvesting, obtaining oils during flowering gave a homogeneous oil content as well as after flowering (Table 4).

In the case of extracting oils from the leaves, the best method of obtaining compounds such as camphene, 1.8-Cineole, and camphor was the HD method, while in the case of borneol and borneol acetate content, the SD method was the best. Moreover, in the case of obtaining 1.8-Cineole, HD and SD methods turned out to be homogeneous in terms of the value of this feature (Table 4).

The comparison of these results with those reported in the literature showed some differences vis-à-vis the major components of the essential oil as well as in the different constituents identified. Dob et al. [14] found that the *Thymus algeriensis* essential oil was dominated by oxygenated monoterpenes, which was followed by monoterpenes hydrocarbons, sesquiterpenes, hydrocarbons, and oxygenated sesquiterpenes, where linalool and thymol were found as major constituents of this essential oil. The α-pinene was identified as the minor compound in this study (3.74%). These results disagreed with those reported by Ali et al. [27], who detected the presence of α-pinene as the major compound (20.50%). The qualitative and quantitative analysis results of *Thymus algeriensis* essential oil are similar to those marked by Hamdani et al., [29] and Nacim et al. [30], with a small variation in the major compound content. Thymol and carvacrol were identified as major compound by Milos [17]; those compounds were absent in the studied essential oil. The differences in the oil quantity may be attributed to the harvest time, the distillation mode, and the geographic and climatic factors.

### 2.2. The Biological Assessment

#### 2.2.1. DPPH and ABTS Radical Scavenging Activity test

The results of the scavenging activity of the studied oil are shown in Table 5, Table 6, Table 7, Table 8, Table 9, Table 10, Table 11 and Table 12. The results of the scavenging of the base oil obtained from the hydrodistillation of the leaves were as follows: DPPH IC_50_ was 8.34 mg·mL^−1^ (Table 5), and the equivalent antioxidant capacity vitamin E (VEAC) was equal to =1.79 mg VE/gEOs; as for the essential oil obtained from the aerial part, IC_50_ with DPPH 41.09 mg·mL^−1^ (Table 8) and equivalent mass 0.657 mgVE/gEOs and IC_50_ with ABTS 10.84 mg·mL^−1^ (Table 9); the equivalent mass here is VEAC = 3.487 mgVE/gEOs, and according to the equivalent antioxidant capacity results, it appears that the leaves essential oil has the greatest scavenging activity. The results shown in Table 4, Table 6 and Table 7 were used to compare the strength of essential oils’ activity with reference compounds known for their strong antioxidant activity. The results of these tables were also used to calculate the equivalent antioxidant capacity of the essential oil per gram of the reference compound. These results clearly demonstrated the moderate antioxidant activity of the *Thymus algeriensis* oil. The results obtained on IC50 values (8379 mg/mL) are consistent with those of Ali et al. [27]. In agreement with Hazzit et al. [26] (Table 2), it is difficult to compare the IC50 values that we obtained with the results of previous publications for the same plant due to the different conditions of each experiment and the concentrations and volumes used. The antioxidant activity of essential oil was claimed to be attributed to the presence of phenolic constituents in plant oils, especially thymol and/or carvacrol as major compounds. However, those compounds are absent in the studied essential oil. The results imply that other non-phenolic compounds are responsible for this activity.

#### 2.2.2. Antimicrobial Susceptibility Assay

Essential oil shows good efficacy against aureus bacteria, which are among the most common pathogens encountered in clinical practices, and they are a major cause of hospital infection, food poisoning, osteomyelitis, endocarditis, toxic shock syndrome, and a wide range of other disorders. It is effective against the bacteria *Escherichia coli*, which is widespread in spoiled foods, causing intestinal disturbances and severe diarrhea. This oil showed statistically significant efficacy against Escherichia coli, while Amoxycyclin had the opposite effect (Table 13).

The essential oil also shows moderate to strong effectiveness on yeasts and fungi, and its effectiveness is stronger than the effectiveness of the reference medicine used (Table 14).

Essential oil significantly limited *Candida tropicalis*, *Candida albicans* came second, and *Candida glabrata* and *Saccharomyces cerevisiae* were in the same homogeneous group in terms of antimicrobial properties. For comparison, the reference drug (Itraconazole) was best limited by *Saccharomyces cerevisiae*, and the worst was *Candida albicans* (Table 14).

#### 2.2.3. Cytotoxic Activity Test

**Figure 2:**
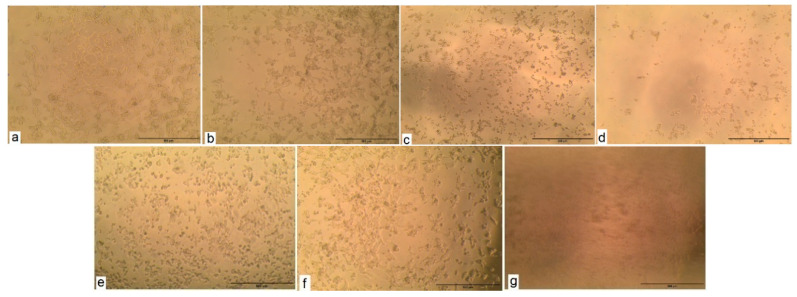
Cytotoxic activity against HCT116 cell line. (**a**): HCT116-*E. oil*—12.5 μg/mL. (**b**): HCT116-*E. oil*—25 μg/mL. (**c**): HCT116-*E. oil*—50 μg/mL. (**d**): HCT116-*E. oil*—100 μg/mL. (**e**): HCT116 control before treatment. (**f**): HCT116- DMSO at 100 ppm. (**g**): HCT116-positive (Doxorubicin) at 100 μg/mL.

**Figure 3:**
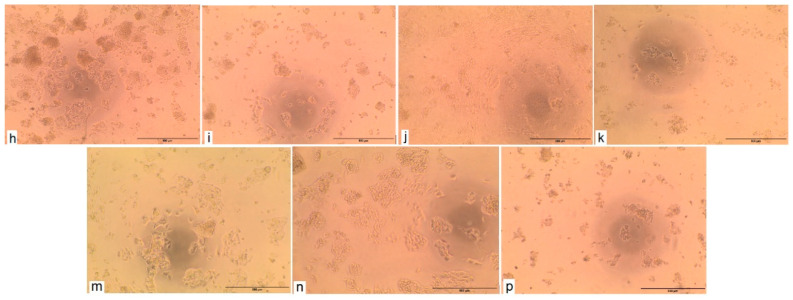
Cytotoxic activity against HePG2 cell line. (**h**): HePG2- *E. oil*—12.5 μg/mL. (**i**): HePG2- *E. oil*—25 μg/mL. (**j**): HePG2- *E. oil*—50 μg/mL. (**k**): HePG2- *E. oil*—100 μg/mL. (**m**): HePG2 control before treatment. (**n**): HePG2—DMSO at 100 ppm. (**p**): HePG2—positive (Doxorubicin) at 100 μg/mL.

The cytotoxic activity test (in vitro bioassay on human tumor cell lines) was conducted and determined by the Bioassay-Cell Culture Laboratory, National Research Centre, Cairo, Egypt. The cytotoxic activity of *Thymus algeriensis* essential oil was tested on the two cell lines: HCT116 (colon cell line) and HePG2 (human hepatocellular carcinoma cell line) (Table 13). The cytotoxicity evaluation was performed for sample concentrations of essential oil that ranged between 12.5 and 100 μg/mL using MTT assays. The results are shown in Table 15. The essential oil possesses a cytotoxic activity on HCT116. As a consequence, the LC50 (lethal concentration of E. oil that causes the death of 50% of cells in 48 h) and LC90 (lethal concentration of E. oil that causes the death of 90% of cells in 48 h) were found to be 39.8 and 59.6 μg/mL, respectively. The inhibition percentage of this essential oil against HCT116 colon cancer cells lines is 100% for 100 μg/mL (Figure 2); this result shows the good effectiveness of this oil when comparing LC50 between it and the reference compound (Doxorubicin), as it is almost equal to it in effectiveness. On the other hand, from the same Table 13, it could be noticed that the tested oil had limited activity against the HePG2 cell line (Figure 3). The research conducted by Milos Nikolic et al. [17] on the anticancer activity of *Thymus algeriensis* essential oil against HCT116 (colon cell line) showed that the dose of IC50 is 64.13 ± 1.33 μg/mL. In fact, the results obtained on the HCT116 colon cancer cell line were very important compared with the positive control of Adriamycin (Doxorubicin), which is 37.6 μg/mL.

## 3. Materials and Methods

### 3.1. Plant Material

**Figure 4:**
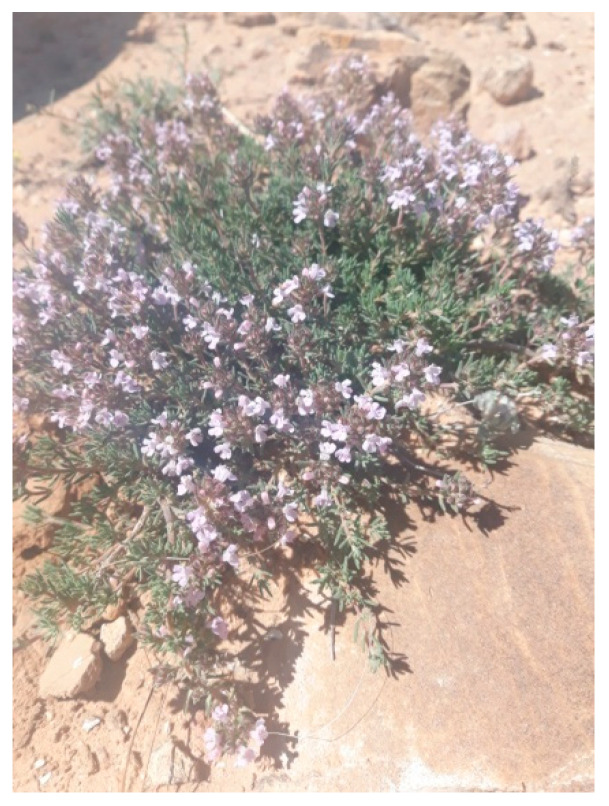
*Thymus algeriensis* Boiss. at flowering stage.

The plant material *Thymus algeriensis* Boiss., which known Himria, was identified by Prof. Naima Benchikha (Chemistry Department, University of Hamma Lakhdar El-Oued, Algeria) (Figure 4). A voucher specimen with a registration number of “RO-040. *T. algeriensis* Boiss.” was deposited with the herbarium of the Chemistry Department, University of Hamma Lakhdar El-Oued. The aerial parts of *Thymus algeriensis* Boiss. Plant (Figure 3) were collected from the El-Guetfa region, M’sila province located in the semi-arid region of Algeria (35°44′26″ N and 3°23′05″ E), during March to June 2018. The number of the gathered samples was selected due to the availability of the plant. The aerial part of the plant (stems, leaves, and flowers) was at first washed with tap and deionized water in order to remove soil particles and dust; next, the samples were air-dried in shade at room temperature over two weeks. Subsequently, the stems, leaves, and flower parts were separated manually and carefully. 

### 3.2. Chemicals and Reagents

The analytical class chemicals, methanol, n-alkanes series (C8-C28), DPPH (2,2-diphenyl-1picrylhydrazyl), ABTS (2,2′-azinobis (3-ethylbenzothiazoline-6-sulfonic acid)), ascorbic acid, trolox (6-hydroxy-2,5,7,8-tetramethylchroman-2 carboxylic acid), butylated hydroxy anisole (BHA), butylated hydroxytoluene (BHT), anhydrous sodium sulfate (Na_2_SO_4_), and potassium persulfate (K_2_S_2_O_8_) were procured from Sigma-Aldrich (St. Louis, MO, USA).

### 3.3. Extraction of the Essential oil (EOs)

At first, preliminary extractions using three distinguished techniques—hydro, steam, and microwaves distillation—were done, subjecting the aerial part of *Thymus algeriensis* Boiss. in order to choose the best technique that showed the highest yield. Next, hydrodistillation was chosen to perform the extraction of the leaves; the extraction process was done after weighting 300 g of the plane leaves directly immersed in the boiling flask of the hydrodistillation system (Clevenger trap). The hydrodistillation process was achieved until there was no significant increase in the volume of the EOs recovered (average of 4 h). The EOs were recovered and dried over anhydrous sodium sulfate (Na_2_SO_4_). Finally, the EOs were kept in a refrigerator at +3 °C in a sealed vial pending analysis. The EO yield was determined as the ratio between the mass of essential oil recovered and the mass of the primary plant material treated.

#### 3.3.1. GC-FID Analysis 

The analysis of the extracted essential oil was performed using an Agilent GC7890a gas chromatography equipped with a flame ionization detector (FID) and a capillary fused bonded column Rtx-5MS (Crossbond diphenyl dimethyl polysiloxane, 30 m × 0.25 mm i.d., 0.25 µm film thickness). The injector and detector temperature were kept at 250 °C and 300 °C, respectively. A volume of 1 μL solution, prepared by 5% vol. EO dilution in n-hexane was injected in split mode (1:50). Nitrogen was used as a carrier gas at a flow rate of 1 mL/min. The column temperature was programmed at 60 °C to 280 °C with an increase increment of 3 °C/min and then left maintained at 280 °C for 5 min. The percentages of the chemical constituents were calculated relative to peaks areas determined by electronic integration of the FID detector.

#### 3.3.2. GC-MS Analysis 

The recovered volatile oils were analyzed by GC-MS, using a Shimadzu GC-MS-QP2010 (Tokyo, Japan) quadrupole mass spectrometer equipped with the same fused bonded column (30 m × 0.25 mm i.d. × 0.25 µm film thickness) used in the GC-FID analysis (Rtx-5MS). The oven temperature was fixed at 45 °C for 2 min; then, it was increased to 300 °C at a rate of 5 °C/min and then left at 300 °C for 5 min. The injection port temperature was 250 °C, while the detector temperature was set at 330 °C, and the injection was done in the split mode with a split ratio of 80:1. The carrier gas used was helium (99.995% purity) with a flow rate of 1.5 mL/min. The mass spectrometer conditions were as follows: ionization voltage 70 eV, ion source temperature 200 °C, and electron ionization mass spectra were acquired over the mass range of 45–500 m/z.

#### 3.3.3. Identification of Chemical Compounds 

LRI “Linear retention indices” were determined for separated compounds relative to a homologous n-alkanes serial (C8–C28). Chemical components were identified by comparison of their calculated LRI with those of literature [31,32], and by evaluation of the mass spectra (MS) with those reported by the NIST and Wiley libraries “NIST & WILEY”. 

### 3.4. Biological Evaluation

#### 3.4.1. Antioxidant Activity

##### DPPH (*1,1*-Diphenyl-*2*-Picrylhydrazyl) Assay

The DPPH scavenging assay has been selected as a primary test to be performed at the initial screening of extracts due to its relatively low cost and the high stability of this chemical regent [33]. The ability of the tested extracts to scavenge the free radical reagent DPPH˙ was assessed according to protocol described by Lue et al. [34]. In test tubes, 1 mL of a fresh methanolic–DPPH solution (250 µM) was vortexed with 1 mL of varied extract concentrations; then, it was incubated at room temperature for half an hour. After incubation, the combination absorbance was calculated against a blank at 517 nm wavelength using a Helios Omega UV/Vis spectrometry. The inhibition percentage was determined using the following Equation (1):

*I%DPPH* = (1 − Abs sample/*Abscontrol*) × 100 (1)

where

− I%: Inhibition percentage (%).
− Abs sample: Absorbance of methanolic-DPPH solution containing extracts after 30 min of reaction time.
− Abs control: Absorbance of methanolic-DPPH solution without extracts.

All analyses were run in triplicates, the activity results were expressed as IC_50_ (g/L) values, ascorbic acid was used as reference in this test.

##### ABTS (2,2′-Azino-Bis (3-Ethylbenzothiazoline-6-Sulphonic Acid)) Test

(ABTS^• +^) radical cation depolarization assay is one of the spectrophotometric analyses extensively used for the estimation of the antioxidant activity. The ability of our extracts (EOs) to scavenge (ABTS^• +^) radicals was achieved according to the protocol described by Rajurkar and Hande [35]. ABTS reagent was prepared and then stored in the dark at room temperature for 12–16 h before use. The next day, ABTS reagent solution was diluted with methanol until we obtained an absorbance value that ranged between 1.03 and 734 nm. Next, 1 mL of the prepared diluted ABTS reagent solution was mixed with 0.3 mL of different EOs concentrations. The mixture was well agitated and incubated in the dark at room temperature for 10 min. Finally, the percentage of inhibition for the extracts was determined using the following Formula (2)

ABTS^•+^ scavenging effect (%) = ((AB − AA)/AB) × 100 (2)

where AB is absorbance of ABTS radical + methanol; and AA is absorbance of ABTS radical sample [35].

#### 3.4.2. Antimicrobial Susceptibility Assay

Three pathogenic bacteria were selected: Micrococcus luteus ATCC 9314, Staphylococcus aureus ATCC 43,300, Escherichia coli (Ec), one Saccharomyces cerevisiae ATCC 4226, three fungi Candida albicans IPA200 (Ca IPA2), Candida tropicalis (Ct), and Candida glabrata (Cg). These strains of bacteria—Candida tropicalis (Ct), Candida glabrata (Cg), and Escherichia coli (Ec)—belong to the collection of the LBSM laboratory (Laboratory of Biology of Microbial Systems of ENS-Kouba, Algeria). Antimicrobial sensitivity of the three essential oils was assessed by a classic disk.

A diffusion test using 800 μL of suspension containing (10^7–^10^8^) mL^−1^ bacteria was spread over nutrient agar medium. Sterile filter paper discs (6 mm in diameter) were soaked in 80 μL of essential oil diluted 1: 1 (*v*/*v*) with methanol (40 µL/disk) and placed on inoculated plates. Negative control was prepared with the same volume of methanol, and Amoxicillin and Itraconazole (25 µg/disk) were used as the positive control. After incubation for 24 h at 37 °C, the antibacterial activity was assessed by measuring the diameter of the zones of inhibition against the tested microbes.

#### 3.4.3. Cytotoxicity Assay 

The evaluation of the EOs toxicity of human cancer cells was done by the mitochondrial-dependent reduction of yellow (which is the part responsible for the production of energy within the living cell). In this study, two cancer cell lines were examined: colon cancer HCT116 (ATCC^®^ CCL­247™) and Hepatic cancer HePG2 (ATCC^®^ HB-8065™). In this experiment, all investigations were performed at the logarithmic growth phase of the cells, where the cell lines were grown in RPMI 1640 media (L-glutamine augmented with 100 U/mL penicillin, 100 U/mL streptomycin, and 25 µg/mL amphotericin B). Cells were grown and developed at the temperature of 37 °C in a 5% CO_2_ humidified atmosphere The determined cytotoxicity was done in triplicate by using the 3-(4,5-Dimethylthiazol-2-yl)-2,5-diphenyltetrazolium bromide (MTT) cell viability assay; 2 × 104 cells/well of exponentially growing cells of both HePG2 and HCT116 cell lines were seeded in a 96-well plate [5]; then, the cells were cultivated for 48 h and then incubated at concentrations (0.78 to 100 μg/mL) at 37 °C for 48 h; after that, it was incubated for 4 h at a concentration of 0.5 mg/mL MTT; finally, the absorbance was detected at 595 nm with a reference wavelength of 620 nm. Doxorubicin (a type of drug in chemotherapy that slows or stops the growth of cancer cells) was used in this test as a positive control. 

### 3.5. Statistical Analysis

Three repetitions were used for samples, and triplicates were made for each measurement in all tests. Results are expressed as mean values and standard deviations (SD). Statistical calculations were performed using SAS Enterprise 4.2 software [29]. Statistical analyses were based on one-way models of analysis of variance (ANOVA) in laboratory experiments with multiple T-Tukey tests, with the adopted significance level *p* = 0.05. T-Tukey’s multiple comparative tests enable a detailed comparative analysis of means by distinguishing statistically homogeneous groups of means (homogeneous groups), which determine the so-called smallest significant differences of means. In Tukey’s tests, they are designated as HSD (Tukey’s Honest Significant Difference).

As part of the descriptive statistics, the generalized (relative, absolute) coefficients of variation were calculated for each CV variable (coefficient of variation in percentage) or the relative standard deviation. They are a measure of the random variability of the experiments performed. The explanations for all tables contain the most important elements of the analysis of variance, including the calculated probabilities (the so-called *p* value) related to the F (Fisher–Snedecor) test functions used. The calculated *p*-values determine the significance and size of the influence of the studied factors on the differentiation of the results of the analyzed variables, comparing them with the most commonly accepted alpha significance levels (0.05). In the case of cytostaticity, statistical significance was tested between the samples and the negative control (vehicle cells) using the independent t-test samples in the SPSS 11 program. Likewise, a probability analysis of the determination of LC50 and LC90 was performed using the SPSS 11 program [25].

## 4. Conclusions

These experiments revealed that the hydrodistillation (HD) of leaves in the flowering period is the best choice. The results showed especially that oil extracted from local populations of *Thymus algeriensis* is rich in camphor, borneol, camphine, 1,8 cineole, and bordyl acetate, in addition to several other organic compounds. Several biological tests were done, especially the antitumor test against colon cancer cell line HCT116, which is according to our literature survey the first study testing this plant EO against this kind of cell cancer. Furthermore, the results were quite amazing comparing with the reference used in this work, which was “Doxorubicin”; in our next experiments, the isolation of the bioactive compounds and re-testing will be more important.

## Figures and Tables

**Table 1:** The chemical composition of essential oil *Thymus algeriensis* (aerial parts and leaves samples), where RI represents the retention index.

*Thymus algeriensis*				Aerial Parts Parts (Stems, Leaves, and Flowers)			Leaves		
				before Flowering	during Flowering	after Flowering	during the Flowering		
N°	Compounds ^a^	RI ^b^	RI ^c^	HD%	HD%	HD%	MAD%	HD%	SD%
1	Tricyclene	921	918	0.475	0.617	0.866	0.343	0.52	0.27
2	α-Thujene	924	921	0.183	0.210	0.084	0.132	0.1	0.05
3	α-Pinene	932	928	4.562	5.007	2.808	3.027	3.74	1.88
4	Camphene	946	941	10.732	12.784	12.858	8.729	14.88	7.53
5	Thuja-2,4(10)-diene	953	950	-	-	-	-	0.09	0.08
6	verbenene	961	962	0.384	-	0.022	0.363	-	-
7	Sabinene	969	963	-	-	0.226	0.95	0.22	0.15
8	β-Pinene	974	965	1.118	1.218	0.73	0.752	0.83	0.48
9	β-Myrcene (Myrcene)	988	977	1.146	1.014	0.216	0.029	0.42	0.18
10	α-Terpinene	1014	1012	0.271	-	0.303	0.081	0.11	0.06
11	p-Cymene (p-Cymol)	1020	1017	1.177	-	-	0.153	0.15	0.15
12	Limonene	1024	1018	-	-	-	0.909	0.66	0.86
13	1,8-Cineole	1026	1020	5.165	5.940	11.209	7.009	7.88	7.72
14	(Z)-β-ocimene	1032	1026	0.141		0.019	0.093	-	-
15	(E)-β-ocimene	1044	1039	2.151	1.728	0.185	1.669	0.53	0.31
16	γ-Terpinene	1054	1051	0.151	0.111	0.256	0.149	0.19	0.09
17	cis-Sabinene hydrate	1065	1061	0.78	0.656	0.827	0.824	0.35	0.97
18	cis-Linaloloxide (furanoid)	1067	1068	0.061	0.134	0.107	0.048	0.06	0.05
19	Camphenilone	1078	1081	0.132	0.235	1.103	0.199	0.3	0.44
20	α–Tterpinolene (Tterpinolene)	1086	1086	0.566	0.205	0.259	0.253	0.17	0.12
21	trans-Sabinene hydrate	1098	1099	2.402	0.866	0.929	2.007	0.83	0.88
22	Linalool	1095	1105	0.193	1.030	0.18	0.033	-	-
23	Isopropyl-5-methyl-(2E)-hexenal<2->	1104	1108	-	-	-	-	0.08	0.21
24	Cis-p-menth-2-en-1-ol	1118	1124	0.41		0.499	0.369		
25	α—Campholenal	1122	1128	0.069		0.051	0.091	0.31	0.33
26	Mentha-2,8-dien-1-ol<cis-p->	1133	1138	-	-	-	-	0.05	0.13
27	Nopinone	1135	1140	-	-	-	0.086	-	-
28	trans-Pinocarveol	1135	1142		-	-	0.239	0.15	0.19
29	Camphor	1141	1145	17.452	22.602	34.31	20.738	32.56	24.25
30	Camphene hydrate	1145	1146	-	-	0.091	0.046	-	-
31	Borneol	1165	1165	13.907	11.164	14.479	16.741	17.13	22.2
32	Terpinene-4-ol	1174	1174	0.56	0.704	1.113	0.761	1.05	0.8
33	p-Cymen-8-ol	1179	1181	0.049	0.104	0.207	0.047	-	-
34	α–Terpineol	1186	1186	0.583	0.489	0.342	0.631	0.33	0.49
35	Myrtenol	1195	1192	0.224	0.214	0.341	0.249	0.17	0.3
36	Verbenone	1204	1202	0.046	0.053	0.016	0.032	0.09	0.2
37	trans-Carveol	1215	1213	0.127	0.139	0.213	0.132	-	0.13
38	Isobornyl formate	1235	1223	0.18	0.137	0.59	0.195	0.15	0.39
39	Neral (Z-Citral)	1235	1239	0.075	0.079	0.136	0.239	-	-
40	Carvone	1239	1241	-	-	-	-	-	0.08
41	Piperitone	1249	1249	1.89	-	0.245	0.085	-	-
42	Geraniol	1249	1251	-	1.361	-	1.913	0.32	0.16
43	Geranial(E-Citral)	1264	1265	0.279	0.258	0.074	0.33	-	0.03
44	Perilla aldehyde	1269	1268	-	-	-	-	-	13.21
45	Bornyl acetate	1284	1281	3.859	3.860	4.278	5.707	5.21	7.92
46	α–Copaene	1374	1372	-	-	0.029	0.056	0.04	0.04
47	β-Bourbonene	1387	1371	-	-	-	0.093	1.3	0.83
48	Geranyl acetate	1379	1370	4.263	2.649	0.206	3.781	-	-
49	β-Elemene	1389	1377	0.408	0.253	0.07	0.268	0.12	0.12
50	α–Gurjunene	1409	1392	0.554	0.279	0.088	0.232	0.21	0.26
51	(E)-β–Caryophyllene	1417	1401	0.123	0.103	0.022	0.068	-	0.05
52	α–Humulene	1452	1447	-	-	-	0.025	-	-
53	AROMADENDRENE	1439	1449	0.717	0.564	0.176	-	-	
54	Alloaromadendrene	1458	1455	-	-	-	0.416	0.39	0.49
55	Germancrene D	1480	1478	-	-	-	-	0.08	0.08
56	α–Amorphene	1483	1473	0.521	0.300	0.05	0.288	-	-
57	Valencene	1496	1484	0.114	0.146	0.046	0.041		0.11
58	Bicyclogermacrene	1500	1491	1.583	1.042	0.219	0.762	0.53	0.6
59	α-Muurolene	1500	1495	-	0.101	0.012	0.018	-	-
60	α-Bulnesene	1509	1499	0.407	0.334	0.162	0.277	0.25	0.35
61	γ-Cadinene	1513	1509	0.158	0.182	0.038	0.107	0.12	0.09
62	Cis-dihydroagarofuran	1519	1515	0.59	0.351	0.113	0.681	-	-
63	δ-Cadinene	1522	1519	0.489	0.724	0.095	0.497	0.56	0.22
64	α-Agarofuran	1548	1544	0.386	0.274	0.209	0.061	-	-
65	Bourbonanone<1-nor->	1561	1558	-	-	-	-	0.22	0.04
66	Palustrol	1567	1562	0.118	0.126	0.1	0.103	-	-
67	Germancrene D-4-ol	1574	1570	1.179	1.195	1.189	1.209	-	-
68	Spathulenol	1577	1572	0.858	0.844		0.231	-	-
69	Caryophyllene oxide	1582	1578	0.161	0.276	0.264	0.035	-	-
70	Capaen-4-α-ol<α->	1590	1588	-	-	-	-	0.9	0.58
71	Viridiflorol	1592	1590	-	-	-	-	0.27	0.16
72	Ledol	1602	1597	0.242	0.306	0.26	0.264	-	-
73	Isolongifolan-7-α-ol	1618	1615	-	-	-	-	0.19	0.1
74	t-Cadinol (α-epi- Cadinol)	1638	1635	0.331	0.864	0.102	0.169	-	-
75	α-epi- Muurolol (tau-Muurolol)	1640	1642	0.383	0.530	0.259	0.363	-	-
76	Muurolol<α->	1644	1645	-	-	-	-	0.31	-
77	α-Cadinol	1652	1653	4.139	2.166	1.634	0.224	-	-
78	Eudesmpl<α->	1652	1657	-	-	-	-	0.12	0.07
79	Alloimachalol	1661	1659	-	-	-	-	0.43	0.09
80	7-epi-α-Eudesmol	1662	1661	-	2.630	-	4.129	2.14	0.89
81	Cedranol<5-neo->	1684	1682	-	-	-	6.034	1.16	0.59
82	Acorenone	1692	1686	8.032	5.844	1.248	-	-	-
83	Acorenone B	1697	1695	0.389	0.425	0.256	0.404	-	-
84	Nootkatol	1714	1712	0.232	0.251	0.164	0.194	-	-
85	Eudesn-11-en-4-α,6-α-diol	1808	1806	-	0.314	0.083	0.274	-	-
**Total Identified**				97.877	95.992	97.266	97.687	98.97	99.050
**Yield of Essential Oil (*v*/*w*) %**				1.20	1.30	0.84	1.07	1.53	1.08
**monoterpene hydrocarbons**				26.90	25.59	21.37	20.91	23.93	14.32
**oxygenated monoterpenes**				40.56	43.34	63.93	49.57	60.34	70.66
**sesquiterpene hydrocarbons**				5.07	4.03	1.01	3.15	3.60	3.24
**oxygenated sesquiterpene**				17.04	16.40	5.88	14.38	5.52	2.48
**other compounds**				8.30	6.65	5.07	9.68	5.58	8.35

^a^: Compounds are identified based on the comparison of their mass fragmentation pattern, and their retention indexes and the compounds were listed in the order of elution from Rtx-5MS gas chromatography column; ^b^: Retention index from literature; ^c^: Experimentally determined retention index (RI); (-): not found.

**Table 2:** Descriptive statistics for the characteristics of essential oils of *Thymus algieriensis.*

Specification	x_1_	x_2_	x_3_	x_4_	x_5_	x_6_	x_7_	x_8_
Average	1315.61	1313.09	1.15	1.13	1.16	1.15	1.16	1.17
Median	1264.00	1265.00	0.18	0.21	0.10	0.20	0.11	0.09
Standard deviation	249.87	249.18	2.87	3.15	4.40	3.18	4.33	3.98
Kurtosis	−1.36	−1.33	17.41	28.22	40.87	23.24	35.86	22.71
Skewness	0.09	0.09	4.00	4.94	6.01	4.58	5.68	4.67
Range	887.00	888.00	17.45	22.60	34.31	20.74	32.56	24.25
Minimum	921.00	918.00	-	-	-	-	-	-
Maximum	1808.00	1806.00	17.45	22.60	34.31	20.74	32.56	24.25
Coefficients of variation (%)	18.99	18.98	248.99	279.32	379.68	277.13	372.15	341.50

x_1_—Retention Index from literature (RI); x_2_—Experimentally determined retention index (RI); x_3_—HD before flowering of aerial parts parts; x_4_—HD during flowering of aerial parts parts; x_5_—HD after flowering of aerial parts parts; MAD during of the flowering of leaves; x_6_—HD during of the flowering of leaves; x_7_—SD during of the flowering of leaves; x_8_—HD after flowering of leaves.

**Table 3:** Pearson simple correlation coefficients for the methods and methods of obtaining thyme oils.

	x1	x2	x3	x4	x5	x6	x7	x8
x1	1.00 *							
x2	1.00 *	1.00 *						
x3	−0.15	−0.15	1.00 *					
x4	−0.16	−0.16	0.96 *	1.00 *				
x5	−0.19	−0.19	0.89 *	0.96 *	1.00 *			
x6	−0.15	−0.15	0.89 *	0.91 *	0.92 *	1.00 *		
x7	−0.19	−0.19	0.90 *	0.96 *	0.99 *	0.95 *	1.00 *	
x8	−0.18	−0.18	0.82 *	0.83 *	0.87 *	0.89 *	0.89 *	1.00 *

x_1_—Retention index from the literature (RI); x_2_—Experimentally determined retention index (RI); x_3_—HD before flowering of aerial parts; x_4_—HD during flowering of aerial parts; x_5_—HD after flowering of aerial parts; MAD during of the flowering of leaves; x_6_—HD during of the flowering of leaves; x_7_—SD during of the flowering of leaves; x_8_—HD after flowering of leaves; * significant at *p*≤0.05; r xy = 0 when there is no correlation; rxy = 1.00, when functional dependence (perfect correlation); 0 < r xy > 1, when the correlation is positive, i.e., with the increase in the value of one variable, the values of the other variable increase and vice versa; >0.3 xy r when the correlation is not clear; 0.3–0.5 xy r when the correlation is mean; >0.5 xy r when the correlation is clear.

**Table 4:** Major compounds of essential oil Thymus algeriensis (%).

Experimental Factors			Camphene	1,8-Cineole	Camphor	Borneol	Bornyl Acetate
Our data Aerial parts	Before flowering	HD	10.732 ^b^	5.165 ^c^	17.452 ^c^	13.907 ^b^	3.859 ^b^
	During flowering	HD	12.784 ^a^	5.940 ^b^	22.602 ^b^	11.164 ^c^	3.860 ^b^
	After flowering	HD	12.858 ^a^	11.209 ^a^	34.310 ^a^	14.479 ^a^	4.278 ^a^
HSD_0.05_			0.670	0.412	1.363	0.731	0.220
Our data leaves	Leaves during the flowering	MAD	8.729 ^b^	7.009 ^b^	20.738 ^c^	16.741 ^b^	5.707 ^b^
		HD	14.880 ^a^	7.880 ^a^	32.561 ^a^	17.130 ^b^	5.210 ^c^
		SD	7.531 ^c^	7.720 ^a^	24.25 ^b^	22.200 ^a^	7.92 ^a^
HSD_0.05_			0.410	0.408	1.423	1.035	0.354
Algeria [26]		HD%	2.3	6.5	10.1	1.6	0.7
Tunisia [16]		HD%	1.83	19.96	19.2	7.64	11.67
Tunisia [27]		HD%	5.9	7.4	14.8	1.2	1.3
Morocco [28]		HD%	0.07	7.64	27.7	2.53	0
Morocco [29]		HD%	11.8	4.9	10	18.3	1.2

Letter indicators at averages determine the so-called homogeneous groups (statistically homogeneous). The occurrence of the same letter for averages (at least one) means that there is no (no) statistically significant difference between them. The sizes of HSD perform an auxiliary role, allowing quantification of the differences between means in a quantitative way.

**Table 5:** Descriptive statistics of the DPPH radical scavenging effect of *Thymus algeriensis* leaf essential oil.

Specification	C (mg/mL)					
	0.4	0.8	1	10	50	150
Average	13.90	20.78	29.36	54.46	66.72	87.14
Median	13.87	20.93	29.14	54.66	67.52	87.34
Standard deviation	0.37	0.51	0.46	0.44	1.41	0.37
Skewness	0.37	−1.21	1.66	−1.63	−1.73	−1.72
Range	0.73	0.99	0.84	0.80	2.46	0.66
Minimum	13.55	20.21	29.05	53.96	65.09	86.71
Maximum	14.28	21.20	29.89	54.76	67.55	87.37
Coefficient of variation V (%)	2.63	2.46	1.57	0.80	2.12	0.43

**Table 6:** DPPH radical scavenging activity of standard solution (experiment with leaves of the plant).

		Concentration (µg/mL)							
Specification		5	10	25	50	100	200	500	IC_50_ (µg/mL)
**I(%)**	**BHT**	0.5 ^c^ ± 0.001	1.0 ^b^ ± 0.003	3.4 ^c^ ± 0.002	7.8 ^c^ ± 0.008	18.5 ^c^ ± 0.002	51.3 ^c^ ± 0.004	67 ^b^ ± 0.007	180.0 ^a^
	**Vitamin E**	15.4 ^a^ ± 0.016	31.0 ^a^ ± 0.002	79.5 ^a^ ± 0.008	89.9 ^a^ ± 0.0014	93.1 ^a^ ± 0.0014	91.1 ^a^ ± 0.008	100.0 ^a^	15.0 ^c^
	**BHA**	13.9 ^b^ ± 0.28	32.0 ^a^ ± 2.98	53.0 ^b^ ± 0.92	78.1 ^b^ ± 1.42	83.7 ^b^ ± 1.49	84.4 ^b^ ± 0.28	10.00 ^a^	22.5 ^b^
	**HSD**_**0.05**_	0.8	1.66	4.21	4.9	4.7	5.2	5.5	10.4

Letter indicators at averages determine the so-called homogeneous groups (statistically homogeneous).

**Table 7:** Descriptive statistics of the DPPH radical scavenging activity of the standard solution (experiment with leaves).

Specification	Concentration (µg/mL)							
	5	10	25	50	100	200	500	IC_50_
Average	9.93	21.33	45.30	58.60	65.10	75.60	89.00	72.50
Standard error	2.37	5.10	11.15	12.82	11.73	6.15	5.50	26.90
Median	13.86	30.69	52.98	78.02	83.09	84.33	100.00	22.50
Standard deviation	7.11	15.31	33.46	38.45	35.19	18.45	16.50	80.69
Kurtosis	−1.71	−1.71	−1.72	−1.72	−1.71	−1.71	−1.71	−1.71
Skewness	−0.82	−0.82	−0.43	−0.79	−0.80	−0.76	−0.86	0.85
Range	14.91	33.98	76.11	82.11	74.60	39.81	33.01	165.00
Minimum	0.50	1.00	3.40	7.79	18.50	51.30	66.99	15.00
Maximum	15.41	34.98	79.51	89.90	93.10	91.11	100.00	180.00
Coefficient of variation V.	71.54	71.77	73.86	65.61	54.06	24.41	18.54	111.30

**Table 8:** Descriptive statistics of DPPH radical scavenging activity of the essential oil of *Thymus algeriensis* (overground part).

	C (mg/mL)					
Specification	16.44	32.87	49.31	65.74	82.18	IC_50_
Average	20.61	41.58	58.41	60.25	65.86	41.09
Median	20.87	41.55	57.66	60.20	66.66	41.09
Standard deviation	1.09	0.97	1.30	1.38	1.56	0.00
Skewness	−1.01	0.14	1.73	0.16	−1.70	0.00
Range	2.14	1.93	2.25	2.75	2.80	0.00
Minimum	19.41	40.63	57.66	58.90	64.06	41.09
Maximum	21.55	42.56	59.91	61.65	66.86	41.09
Coefficient of variation V.	5.31	2.32	2.22	2.28	2.37	0.00

**Table 9:** DPPH radical scavenging activity of standard solution (Vitamin E) (experiment with the aerial part of the plant).

Specification	Concentration (µg/mL)					
	4.60	9.20	13.80	18.40	23.00	27.60
Average	0.48	12.94	20.86	26.73	35.49	52.27
Median	0.49	12.88	20.68	26.77	35.02	52.13
Standard deviation	0.03	0.85	0.85	1.08	0.96	1.45
Skewness	−1.46	0.32	0.91	−0.17	1.68	0.43
Range	0.05	1.70	1.68	2.16	1.73	2.88
Minimum	0.45	12.12	20.11	25.63	34.86	50.90
Maximum	0.50	13.82	21.79	27.79	36.59	53.78
Coefficient of variation V.	5.51	6.58	4.10	4.04	2.69	2.76

**Table 10:** DPPH radical scavenging activity of standard solution (BHA) (experiment with the aerial part of the plant).

Specification	Concentration (µg/mL)			
	2.71	5.42	10.83	60.00
Average	25.08	44.21	62.31	93.69
Median	24.99	44.03	62.62	93.04
Standard deviation	0.74	0.88	1.00	1.21
Skewness	0.54	0.88	−1.26	1.72
Range	1.47	1.74	1.93	2.15
Minimum	24.39	43.43	61.19	92.94
Maximum	25.86	45.17	63.12	95.09
Coefficient of variation V	2.95	2.00	1.61	1.30

**Table 11:** ABTS radical scavenging activity of leaves of *Thymus algeriensis* essential oil.

Specification	Concentration (µg/mL)			
	2.58	9.54	19.08	28.62
Average	30.77	52.31	61.54	90.77
Median	30.73	52.29	60.77	90.55
Standard deviation	0.78	1.19	1.47	2.00
Skewness	0.23	0.08	1.71	0.49
Range	1.56	2.38	2.63	3.98
Minimum	30.01	51.13	60.61	88.89
Maximum	31.57	53.51	63.24	92.87
Coefficient of variation V	2.54	2.28	2.40	2.20

**Table 12:** ABTS radical scavenging activity of standard solution of vitamin E.

Specification	Concentration (µg/mL)			
	6.22	22.97	45.95	68.92
Average	11.97	32.12	59.85	81.06
Median	12.12	32.01	60.01	82.19
Standard deviation	0.35	0.85	1.83	2.08
Skewness	−1.57	0.57	−0.39	−1.72
Range	0.65	1.69	3.64	3.67
Minimum	11.57	31.33	57.95	78.66
Maximum	12.22	33.02	61.59	82.33
Coefficient of variation V	2.92	2.65	3.05	2.57

**Table 13:** Antimicrobial (Bacteria) activity of the essential oil using disc diffusion.

The Bacteria Strain	Diameter of Inhibition Zones (mm)	
	Essential Oil (40 µL/Disk)	Amoxicillin (25 µg/Disk)
*Micrococcus luteus (Ml)*	18.0 ^a^ ± 0.6	0.0
*Staphylococcus aureus CIP 7625*	18.0 ^a^ ± 0.7	10.0 ^b^ ± 0.5
*Escherichia coli ATCC 10536*	13.0 ^b^ ± 0.9	27.0 ^a^ ± 0.7
HSD_0.05_	1.0	1.5

Letter indicators at averages determine the so-called homogeneous groups (statistically homogeneous).

**Table 14:** Antimicrobial (yeasts and fungi) activity of the essential oil using disc diffusion.

The Yeasts and Fungi Strain	Diameter of Inhibition Zones (mm)	
	Essential Oil (40 µL/Disk)	Itraconazole (25 µg/Disk)
*Saccharomyces cerevisiae ATCC 4226*	17.0 ^b^ ± 0.5	0.0 ^d^
*Candida albicans IPA200*	13.0 ^c^ ± 0.4	24.0 ^a^ ± 0.9
*Candida tropicalis (Ct)*	2.04 ^d^ ± 0.8	17.0 ^b^ ± 0.7
*Candida glabrata (Cg)*	18.0 ^a^ ± 0.6	13.0 ^c^ ± 0.4
HSD_0.05_	1.1	1.3

Letter indicators at averages determine the so-called homogeneous groups (statistically homogeneous).

**Table 15:** Cytotoxic activity of *Thymus algeriensis* essential oil against human cell lines (% of inhibition cells ± SEM).

Sample Code (Cell Line)	Concentration (µg/mL)				LC50 (μg/mL)	LC90 (g/mL)	Doxorubicin LC50 (μg/mL)	DMSO at 100 ppm
	100	50	25	12.5				
HCT116	100 ^a^	71.37 ^a^	22.36 ^a^	4.77 ^a^	39.8	59.6	37.6	1%
HePG2	44.2 ^b^	0.0 ^b^	0.0 ^b^	0.0 ^b^	>100	>100	21.6	1%
HSD_0.05_	5.5	4.0	1.3	0.3				

Letter indicators at averages determine the so-called homogeneous groups (statistically homogeneous). Every value represents the mean percentage of inhibition cells of three replicates ±SEM (mean of standard error). LC_50_: Lethal concentration of the sample that causes the death of 50% of the cells in 48 h. LC_90_: Lethal concentration of the sample that causes the death of 90% of the cells in 48 h. HePG2 (human hepatocellular carcinoma cell line) HCT116 (colon cell line).

## Data Availability

All data generated or analyzed are contained within the present article.
